# IQM: An Extensible and Portable Open Source Application for Image and Signal Analysis in Java

**DOI:** 10.1371/journal.pone.0116329

**Published:** 2015-01-22

**Authors:** Philipp Kainz, Michael Mayrhofer-Reinhartshuber, Helmut Ahammer

**Affiliations:** Institute of Biophysics, Center for Physiological Medicine, Medical University of Graz, Graz, Austria; UGent / VIB, BELGIUM

## Abstract

Image and signal analysis applications are substantial in scientific research. Both open source and commercial packages provide a wide range of functions for image and signal analysis, which are sometimes supported very well by the communities in the corresponding fields. Commercial software packages have the major drawback of being expensive and having undisclosed source code, which hampers extending the functionality if there is no plugin interface or similar option available. However, both variants cannot cover all possible use cases and sometimes custom developments are unavoidable, requiring open source applications. In this paper we describe IQM, a completely free, portable and open source (GNU GPLv3) image and signal analysis application written in pure Java. IQM does not depend on any natively installed libraries and is therefore runnable out-of-the-box. Currently, a continuously growing repertoire of 50 image and 16 signal analysis algorithms is provided. The modular functional architecture based on the three-tier model is described along the most important functionality. Extensibility is achieved using operator plugins, and the development of more complex workflows is provided by a Groovy script interface to the JVM. We demonstrate IQM’s image and signal processing capabilities in a proof-of-principle analysis and provide example implementations to illustrate the plugin framework and the scripting interface. IQM integrates with the popular ImageJ image processing software and is aiming at complementing functionality rather than competing with existing open source software. Machine learning can be integrated into more complex algorithms via the WEKA software package as well, enabling the development of transparent and robust methods for image and signal analysis.

## Introduction

Researchers are often confronted with situations, where data needs to be analyzed quickly in a preferably easy way. Commercially available software packages may not meet current requirements in an out-of-the-box configuration, are not available or license fees are too expensive. Furthermore, source code is often undisclosed and there is no opportunity to change or adapt parts of existing applications. This is a commonly known issue and researchers have to write their own code for each specific problem [1].

Due to these drawbacks of commercial software, the number of open source users has increased during the last decade. More permissive licenses and the advantage of cross-platform availability contribute to the considerable increase in open source users, who can now choose from a broader variety of software to fit their particular use cases and enhance the outcome of their research [2].

One important aspect in increasing productivity is having an existing framework that already provides functionality for common and frequent actions like loading and saving relevant file formats. Such tasks, however, are not directly related to the scientific or algorithmic problem, but may hamper progress tremendously.

These facts gave reason to the development of IQM, which has initially been developed by one of the authors in 2004 using IDL (Interactive Data Language, Exelis VIS, Boulder, Colorado, USA) programming language. Several image processing algorithms have been implemented and in 2009 IQM has been migrated to the Java platform [3]. Especially with the analysis of images using fractal dimensions [4] the visualization requirement for multi-dimensional results emerged. As a consequence thereof, basic signal analysis algorithms and plotting were added in late 2012. In recent months, several contributors have implemented and tested algorithms for image and signal analysis in the IQM framework. The current stable version is 3.2 and the project is published under the GNU General Public License version 3 on the open source hosting platform sourceforge.net [5].

Open source software has several advantages for scientific research: it is free, well reviewed by the community and most of the time extensible for custom requirements. There are several open source applications for image analysis, which will be listed in the next section. IQM provides some unique characteristics for image and signal analysis, which are presented in this paper.

### Organization of this Paper

After giving a brief introduction to the field of open source research software and stating the rationale behind the development of IQM, the remainder of this paper is organized as follows.

We review existing open source applications for image analysis and name the intended audience of this paper in the current section. Furthermore, a brief installation guide is presented here. Within the subsequent sections we give an overview of IQM’s key components, briefly describe the functionality, and both the system and functional architecture. After that, a proof-of-concept analysis is presented, demonstrating how users can benefit from the unique characteristic of combined image and signal processing in a single portable open source tool.

We demonstrate, how the IQM framework can be extended via operator plugins and how it integrates with standard open source software for image processing and machine learning. The structure of plugins and operators is explained using the example of an image operator plugin. Then, we briefly introduce the scripting interface of IQM and demonstrate its usage in an automated image processing workflow. Eventually, we will discuss possible future developments of IQM and open source research software tools.

This paper is supplemented by the source code used in the operator and script example (S1 File and S2 File), as well as the syntax-highlighted version of the script (S1 Listing). For further details on the sections, we kindly refer the reader to the supporting information as well as the detailed user guide available from the project website http://sf.net/projects/iqm/files/3.2/IQM3.2_userguide.pdf.

### Related Work

In this section we briefly describe several free and open source software packages for image and signal analysis. Our focus is on software that can be applied to similar tasks as IQM, is usually and frequently used in life sciences, and hence is cited in corresponding literature. We raise no claim to completeness of the following overview, a more extensive list can be found e.g. in a review by Eliceiri et al. [1] or at rsb.info.nih.gov/ij/links.html. Open source image processing libraries like OpenCV [6], openIP [7] or the Insight Segmentation and Registration Tookit (ITK) [8] are not included in this list, however, they should be noted in this context.

- *ImageJ*, imagej.nih.gov/ij:
  The popular image processing software *ImageJ* (current version: 1.48t, Java) is well known for its open plugin architecture. It can be extended by developing new plugins or by recording macros, which is both possible for instance with an included editor and a Java compiler. Furthermore, it is possible to run ImageJ not only as an application on a Java virtual machine but also as an online applet [9–11]. Fields of application include, amongst others, different life and medical sciences, as for example (molecular) biology, radiology, and digital pathology [12–14]. At the moment, a completely rewritten version, *ImageJ2*, is under development (see developer.imagej.net/about).
- *Fiji*, fiji.sc:
*Fiji* (current version: Madison, Java) is a distribution of *ImageJ*, which is especially optimized for an application in life sciences and particularly for the analysis of microscopy images. It provides detailed descriptions of the included algorithms, extensive documentations and tutorials with the aim to enhance collaborations between research communities from computer sciences and biology [15].
- *CellProfiler*, www.cellprofiler.org:
  The software *CellProfiler* (current version: 2.1.0, Python) has its main applications in biology and focuses on user-friendly automated measurements of phenotypes, i.e. to extract quantitative information from biological images [16–19]. Additionally, a free and open source software package *CellProfiler Analyst* (current version: 2.0) is available, which may be used for exploration and analysis of large, high-dimensional data, e.g. with several included machine learning tools [20, 21].
- *Icy*, icy.bioimageanalysis.org:
  The bioimage informatics platform *Icy* offers a correspondent software package (current version: 1.5.2.0, Java) for image analysis together with a broad variety of plugins. The main goals of this project are reproducibility, standardization, and easy management of plugins and workflows. It supports graphical programming and script development in Javascript and Python [22].
- *Endrov*, www.endrov.net:
  The image analysis program *Endrov* (current version: 2.23.2, Java) can be used for image acquisition from light and fluorescence microscopes as well as for post-processing. Main features are e.g. the support of high-dimensional microscopy data and the ability for handling large datasets up to sizes over 100 gigabytes [23].
- *BioImageXD*, www.bioimagexd.net:
  The software package *BioImageXD* (current version: 1.0, Python and C++) was developed with a focus on the needs of microscopists and cell biologists. It can be especially used for visualization of multi-dimensional microscopy images, e.g. to create animations of complex 3D renderings by using virtual camera flying paths, and appropriate object-based and voxel-based analysis [24].
- *TMARKER*, www.comp-path.inf.ethz.ch:
  Automated analysis of immunohistochemically stained tissue can be automated with the software toolkit *TMARKER* (current version: v1.20614, Java), which uses machine learning algorithms based on randomized decision trees and support vector machines [25].
- *VisBio*, loci.wisc.edu/software/visbio:
  The software tool *VisBio* (current version: 3.40rc1, Java) can be used for visualization of high-dimensional image data and its analysis. Its special focus is on the processing of large and complex datasets, as they are produced e.g. by newly developed laser scanning microscopy techniques in biology [26, 27].

Although well-established public domain image processing programs such as ImageJ [11] include basic methods for fractal image analysis, e.g. the binary box-counting method, freely available tools for more advanced fractal image and signal analysis are quite rare. However, research groups often publish their implemented algorithms online. In the following an overview over the most popular, freely available fractal analysis packages or plugins is given.

- *FracLac* (*ImageJ*), imagej.nih.gov/ij/plugins/fraclac:
  Developed as a plugin for *ImageJ*, *FracLac* can be used to calculate mass and box-counting dimensions and to perform multifractal and lacunarity analysis of black and white as well as colour images. Suggestions from e.g. the ImageJ, neuroscience, and engineering communities have been included in its development [28].
- *Fractalyse*, www.fractalyse.org:
  The software package *Fractalyse* can be used for fractal analysis of black and white images. In addition to mass and box-counting dimensions, correlation, dilation or Gaussian convolution approaches are implemented [29].
- *FDim*, reuter.mit.edu/software/fdim:
  The fractal dimension of grey value images can be estimated with *FDim*, which supports calculation of the capacity, the information, the correlation and the probability dimensions.
- *Gwyddion*, gwyddion.net:
  The open source software *Gwyddion*, which was developed for scanning probe microscopy, also offers fractal analysis of images. It includes a tool to apply either a box-counting, a triangulation, a variance, or a power spectrum method for the estimation of fractal dimensions [30].

All mentioned software applications can be used without programming skills in a high-level graphical user interface. Some of them also provide command line interfaces for integration in external tools and tasks. However, IQM expresses several characteristics that are not expressed by the mentioned software applications and which we will emphasize throughout the paper wherever appropriate:

1. Combined image and signal analysis from a high level GUI in a single tool,
2. Convenient management of images and signals in a clearly arranged graphical user interface (GUI),
3. Advanced and versatile stack processing of heterogeneous data in serial or parallel mode,
4. Interactive evaluation of image and signal processing algorithms in a “preview” mode,
5. Managing persistent parameter preferences for individual algorithms,
6. Multiple independent image annotation layers,
7. On-demand switching between in-memory and disk-cached (“virtual”) processing modes, and
8. Integrated comprehensive fractal image and fractal signal analysis.

We are not aiming at providing a library of image processing algorithms or substituting any of the mentioned products, but aim at the simple exploration of algorithms in a user-friendly environment, where the results remain reproducible.

### Intended Audience

With this paper the authors want to address researchers and software engineers in life science, medical science, computer science, and natural sciences. We provide a free, extensible, easy-to-use, and cross-platform software application for the exploration and development of image and signal processing algorithms.

Additionally, IQM could be used in higher education courses in order to visualize and demonstrate the basics of simple as well as sophisticated algorithms. These algorithms and concepts can be tested with various parameters using an intuitive GUI even without programming skills. Lecturers are often searching for proper tools for the illustration of essential principles. It would be a tedious task to develop individual isolated applications for each showcase and most of the time, one would have to pay a considerably large license fee for a timely restricted use of commercial products.

With IQM, we tackle these problems by providing an open source and cross-platform software written in one of the leading programming languages for application development. The advantages of IQM are furthermore, that it is freely available and that the software offers a framework for the integration of both image and signal analysis operators.

### Installation

Since IQM is written in pure Java, it remains portable to various operating systems, where a Java Virtual Machine (JVM) exists. The installation of the compiled IQM software package is common to all operating systems and quite straightforward. However, the presence of a JVM is a prerequisite. Packaged binary distributions of IQM can be downloaded from the project website at http://iqm.sf.net. Separate packages are available for different operating systems, where the Java archives have been packaged for system integration in Microsoft Windows, Linux and Mac OS X. After downloading, no explicit installation step is required and the application can be launched immediately in the standard configuration. For more detailed installation instructions the reader is referred to IQM’s user guide.

## Overview and Functions

### User Interface and Main Components

**Figure 1:**
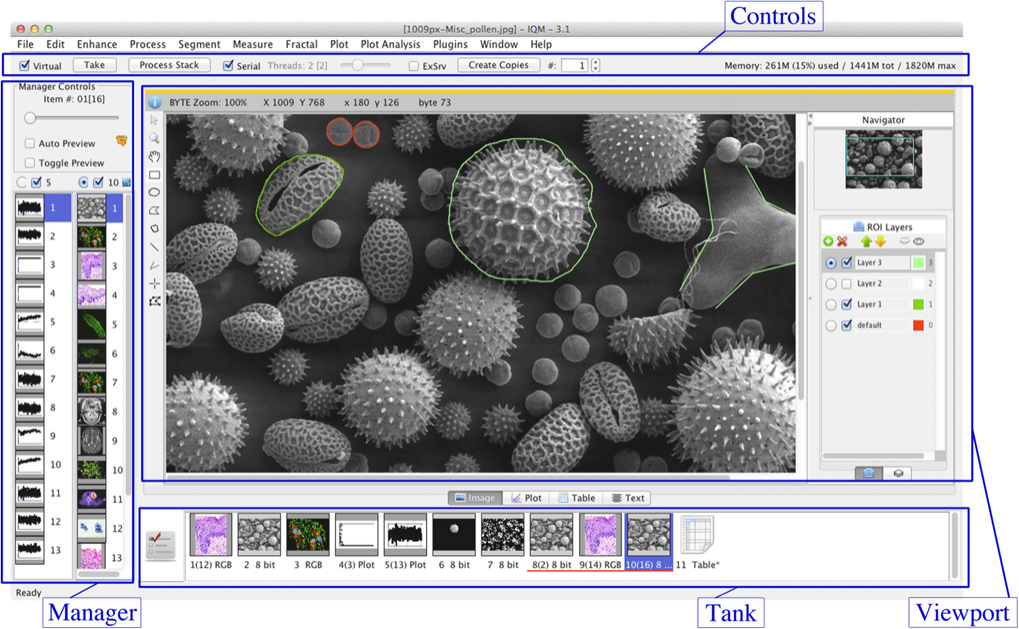
IQM Main Window and Components. The major components Tank, Manager, Viewport and Controls are highlighted. Tank hosts all items (images, signals, tables) and serves as a history of processing steps. The Manager shows thumbnails of the items at the selected Tank index and lets the user select items, which will be displayed in the Viewport. Controls are responsible for setting the application to the “virtual” mode, monitoring the JVM at runtime and determining the stack processing type (serial/parallel).

In order to establish a common understanding of how IQM is used and how the major components interact, we start by describing the main parts of the user interface. The main window hosts the key elements of the GUI: (i) Tank, (ii) Manager, (iii) Viewport and (iv) Controls, see Fig. 1.

Each processing step in IQM starts by putting a single item or an item stack to the Tank. Items may be either images or signal data for subsequent processing, or table data for visualization. They are parsed and loaded from the file system or, in the case of images or signals, can be generated within the application. The Tank can be viewed as container of all loaded items and serves as a kind of processing history. Results of previously executed operators are kept in the list while the user may explore other algorithms or compare different results.

The Manager provides two identically constructed lists and coordinates the visualization and availability of single items or item stacks to operators. Only stacks of homogeneous data type, e.g. images, are permitted to be loaded in the manager lists. A selected radio button on top of the lists indicates the currently active list and hence the primary item source when launching operators. In the context of this paper, the term “operator” is used as synonym for algorithm. All selected items are scheduled for being processed by operators, enabling flexible and selective processing of particular items. Both Tank and Manager are able to merge single items to stacks. Additionally, the Manager can split stacks into single items or do pairwise export from both manager lists.

The Viewport provides four tabs for visualization of images, signals, tables and text. When items are selected, they are immediately visualized in the corresponding tab. Additionally, “preview” results of operators are viewed, which can subsequently be approved and appended to the Tank for further processing. Each tab provides data type specific tools for adjusting the view, or e.g. in case of images to annotate them.

In the Controls section of the main window, the user can approve the preview results of operators, or launch serial or parallel stack processing of items selected in the Manager.

### Digital Image Analysis

Digital image analysis involves image enhancement techniques, conversions between various color and sample models, resampling as well as annotation and segmentation. In this section, we briefly summarize the most important functions and unique features IQM provides for digital image analysis. All implemented image processing operators are enlisted in Table 1, grouped by their main category.

**Table 1:** Implemented Image Processing Operators.

**Main Category**	**Operator Name**
Analysis	Box Dimension* [52, 55–57]
	Complex Logical Depth* [58]
	Fast Fourier Transform* [59, 60]
	Fractal Scan*
	Generalized Dimension*
	Higuchi Dimension* [51]
	Lacunarity*
	Minkowski Dimension*
	Pyramid Dimension*
	Discrete Fourier Transform
	Gradient Vector Field
	Histogram Modification
	Image Calculation
	Intensity Statistics
	Stack Statistics
	Value Calculation
Generator	Fractal Surfaces*
	Iterative Function System*
	Image Generator
Object Detection	Template Matching
Processing	Fractal Surrogates* [61]
	Affine Transformations
	Auto Correlation Function
	Border Extender
	Color Balancer
**Main Category**	**Operator Name**
	Color Space Conversion
	Distance Transform [62]
	Geometric Transformations
	Image Crop
	Intensity Inverting
	Neighbourhood Pixel Scan Rank Filter
	Resize
	Smoothing
	Unsharp Masking
Registration	BUnwarpJ [63]
	Center of Gravity Registration
	Image Stabilizer
	TurboReg [64]
Segmentation	Color Deconvolution
	Edge Detection
	Fuzzy k-means Clustering
	k-means Clustering
	Mathematical Morphology
	Region of Interest Segmentation
	RGB Relative
	Seeded Region Growing
	Statistical Region Merging
	Threshold [65]
	Watershed

This table shows all implemented image operators grouped by their main category. An operator can also contain subroutines which are triggered and parameterized via the graphical user interface. Operators denoted by (*) are fractal operators.

Convenient file input and output is important to any application and hence standard image file formats used in everyday computing are supported. Furthermore, IQM supports some file formats commonly used in (bio)medical imaging. Thus, we are using the functionality of the OME Bio-Formats library [31] and ImageJ plugins to read these formats.

**Image Enhancement, Manipulation and Registration**. If required for specific image calculations or validating algorithms, images can also be artificially generated. The built-in image generator creates 8bit or 16bit grey value images of custom size from different models like random grey values, Gaussian distribution, constant values as well as sine and cosine functions.

IQM offers image enhancement routines for linear and non-linear smoothing and denoising, edge detection algorithms, and a standard deconvolution for sharpening images [32]. Especially for image data obtained via scanning, where noise and distortions of the color spectrum are degrading the image, white and color balance can be used for correcting intensity values. Additionally, image histograms can be modified and standard as well as affine image transformations are available.

Linear transformations of the intensity spectrum using a constant value can be achieved via arithmetic and logical operators. For the segmentation of objects using binary masks or the combination of two images, image calculations are an invaluable tool. Binary masks can be prepared via pre-processing steps like edge detectors and morphological operations (erosion, dilation, …) with various kernel shapes. The mask and the original images may subsequently be combined pixel-by-pixel using arithmetic or logical operators.

Aligning images properly is often a key requirement for image stacks, for instance when tracking objects through a sequence of images, preparing a set of MRI or CT slices for 3D volume rendering or for inter-modality registration. Registering is the process of finding a valid global (rigid) or locally varying (nonrigid/nonlinear) transformation in order to fit one image to another [33]. Simple translations within a stack can be corrected with a center of gravity registration, but warping or other distortions require more sophisticated algorithms. ImageJ already provides a range of plugins for registration and IQM integrates this existing functionality in its framework.

**Segmentation and Texture Analysis**. Segmentation is one of the most important parts in image analysis. Generally, IQM provides intensity-, edge-, and region-based algorithms for segmenting an image. These algorithms can be applied to the image either globally or locally in an interactive manner.

Global statistical texture parameters can be examined using the Grey Level Co-Occurrence Matrix (GLCM). The statistics operator computes statistical properties based on intensities as well as first and second order moments [32] in various directions from the GLCM. Local image textures can be examined in an interactive manner using a sliding window approach.

**Figure 2:**
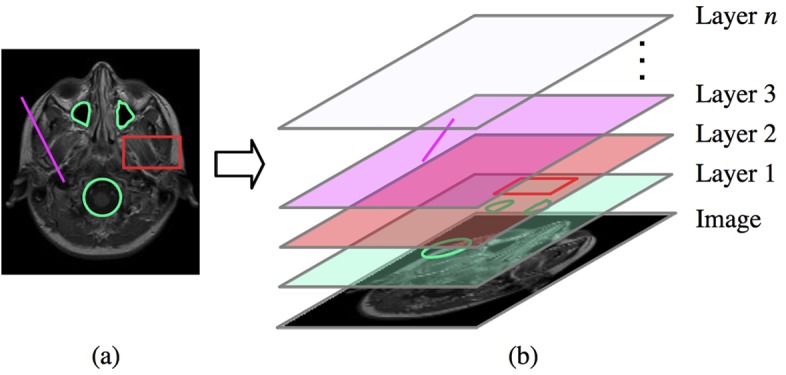
Annotation Layers. Multiple instances of various ROI shapes may be placed on each annotation layer: (a) shows an annotated image and (b) the assignment of the ROI shapes to layers 1–3. The image may be exchanged while keeping the ROIs on each layer.

**Regions of Interest and Annotation Layers**. Images are a multi-dimensional source of information, but when the content is heavily depending on the interpretation, the image alone may often be insufficient. This obstacle can be overcome by adding custom annotations to the image. Tools for drawing and modifying regions of interest (ROIs) are invaluable for custom image annotation. IQM provides various geometric shapes to be used on independent annotation layers, see Fig. 2. Each layer may contain multiple instances of available ROI types. We did not aim at binding ROIs to each image, but to each image canvas. Hence, ROIs do not disappear when the user changes the underlying image. Moreover, scrolling through an image stack always preserves the ROI’s location, since the viewport location does not change either. This facilitates the examination of different images at the same position with the same annotations. Layers are stacked on top of each other, may be reordered, renamed, recolored, displayed or hidden.

The content of annotation layers can be exported to files and imported later on. In automated workflows like scripts this facilitates documentation and reproducibility of results and thus repeatability of entire experiments, which is a key requirement for scientific research. On image export, visible layers may optionally be superimposed on the image, see Fig. 2(a).

### Digital Signal Analysis

In addition to images, i.e. two-dimensional data, IQM allows for the analysis of digital signals, i.e. one-dimensional sequences of numbers. The purpose of this analysis is the extraction of meaningful information from both measured as well as artificially generated signals. Within IQM, signals are treated as one-dimensional sequences of numbers in a dynamical memory structure. Multiple signals can be processed in a serial or parallel manner. In this section, we summarize the most important functions and unique features IQM provides for digital signal analysis. All implemented signal generators and processing operators are enlisted in Table 2, grouped by their main category.

**Table 2:** Implemented Signal Processing Operators.

**Main Category**	**Operator Name**
Analysis	Allometric Scaling*
	Higuchi Dimension*
	Hurst Coefficients*
	Auto Correlation Function
	Fast Fourier Transform
	Period Finder [66]
	Signal Entropy [67–70]
	Signal Statistics [71]
	Symbolic Aggregation [72]
Generator	Fractal Generator* [73]
	Discrete Chaotic Map [74–76]
	Signal Generator
Processing	Fractal Surrogates* [61]
	Filter
	Mathematical Operations
	Signal Extractor

This table shows all implemented signal operators grouped by their main category. An operator can also contain subroutines which are triggered and parameterized via the graphical user interface. Operators denoted by (*) are fractal operators.

One or multiple signals can be loaded from plain text files, lines for header and units are considered and the required data can be selected. Additionally, IQM offers the possibility to generate artificial signals with different signal generators.

Basic manipulation is implemented to allow an extraction of interesting parts of the signal. Moreover, mathematical methods, determination of common statistical parameters, and a period finder for periodic signals are available. IQM also allows surrogate data testing, e.g. to test for non-linear structure in a signal.

### Fractal Analysis of Images and Signals

IQM contains a substantial extension in order to perform fractal and nonlinear analyses of binary as well as grey value images. The main purpose of these methods is texture analysis, particularly useful for natural as well as medical images which often exhibit fractal features [34, 35]. Besides global methods, there is also a local fractal scanning method in order to compute fractal dimensions as local image content descriptors. Signals may also exhibit fractal features, which can be quantified using implemented estimators. For testing purposes, it is also possible to generate images and signals with mathematically known fractal dimensions.

All implemented fractal operators for images and signals are enlisted in Table 1 and Table 2, grouped by their main category.

### Advanced Stack Processing

In the following, we describe a unique characteristic of IQM, the stack processing capability for heterogeneous items. In the context of this paper, we refer to a set or sequence of items (signals or images) as “stack”. Especially in pre-processing, particular homogenization steps like noise filtering or range normalization may be required for numerous items. Unless operations can be applied to each item, parameters of the algorithm must be adjusted. In order to find suitable parameters, the examination of its response across multiple items is essential. This can be done by on-line evaluation of operator settings with immediate feedback using the “preview” functionality, which is another specialty of IQM.

Other open source image analysis applications like ImageJ and its derivatives [11] offer image stack processing as well. However, they impose certain constraints on the nature of images. They have to be exactly of the same size, otherwise loading fails and only images of equal size as the first one are loaded. Another drawback is that each image must be encoded with the same sample and color model (e.g. 8bit single band, or 24bit RGB). This is problematic, since the evaluation of an algorithm with a specific parameter set across multiple images is often required. Challenged by similar situations, an advanced and more flexible stack processing capability has been implemented in IQM. The result of an algorithm can be analyzed with the same parameters across different images in an on-line manner. The size of the single image within the stack does not matter, and—for algorithms that implement adaptive processing—neither does the color or sample model. However, it has to be noted that each algorithm that needs to be applied to heterogeneous image stacks has to implement a compatibility between different color models. If the algorithm does not incorporate that compatibility and the color models vary within a stack, the user has to split it up and merge similar color models again. Furthermore, the zoom factor and the field of view across multiple images remains constant. This feature is particularly useful when the results of different parameter sets or even different algorithms need to be compared at the same magnification and position. Besides images, also signals of various lengths can be processed as stacks.

Once the determination of parameters is satisfactory, the stack may be processed in a serial or parallel manner. The main difference between these processing methods is that parallel processing makes use of multiple CPU cores by distributing the workload to distinct threads. On the other hand, serial processing considers the stack as first-in first-out queue and applies an algorithm to each item sequentially.

IQM’s advanced stack processing enables the analyst to search for suitable algorithm parameters with immediate visual feedback on a high level GUI.

## System Architecture and Functional Components

**Figure 3:**
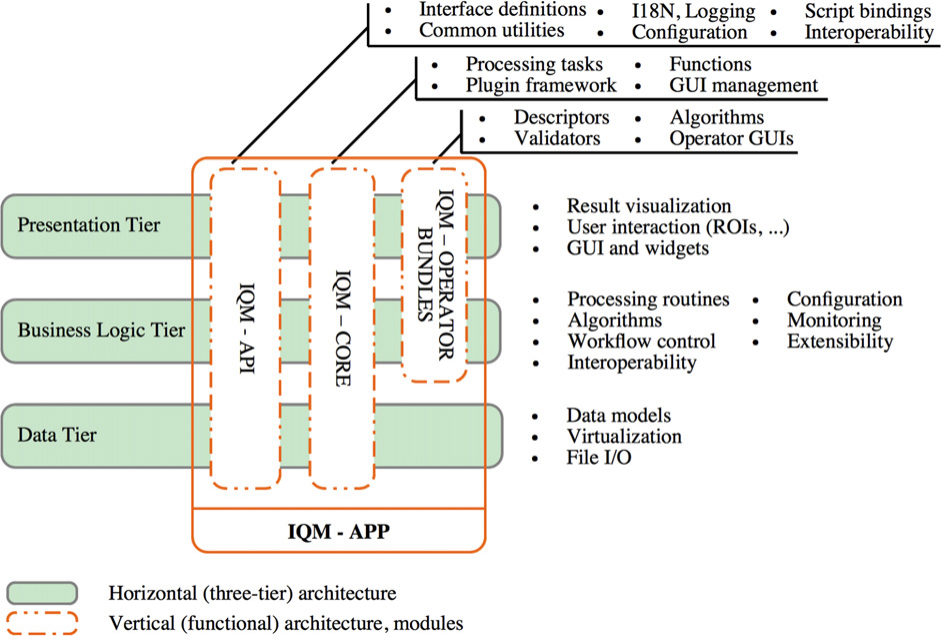
Three-Tier and Functional System Architecture. The system architecture of IQM can be illustrated as classical three-tier model. The functional architecture is composed of different modules and spans vertically over one or more tiers. The “IQM-APP” module ties together all functional components and represents the entire application.

The internal software architecture of IQM may be viewed from both a layer view (horizontal) and a functional view (vertical), see Fig. 3. In this section we briefly want to introduce the system architecture and the functional components and give the reader an idea of how the components are connected and where plugins and scripts fit in.

### Three-Tier Architecture

IQM follows a classic three-tier model of software architecture [36]. Data management is separated from business logic and representation components, facilitating flexibility and extensibility in the development process. The software is designed to be process-oriented.

**Data Tier**. In the context of a running IQM instance all persistent and transient data items, e.g. a single image, a couple of signals, or table objects, are managed by software components attributed to the data tier. These data items are the most basic unit, where operations can be executed on. They may be either loaded to the memory directly or serialized to a temporary directory in order to save memory for the actual processing routine. Thus, the data tier plays an important role in file access tasks by providing functionality for decoding and encoding various contents. Data is loaded and parsed into a flexible internal data model, which manages the original data item as well as its low-level properties such as file names and paths.

Keeping items in memory that are irrelevant to current computations is not efficient, since they are blocking valuable memory. Processing large collections of items may also become quite memory-consuming. Hence, we implemented on-demand serialization or “virtualization”, which is another unique characteristic of IQM. Within the virtualization framework, every data item marked as “virtual” is serialized to a temporary directory conjointly with its meta-data, where it remains accessible to the application. If the application context requires the access of a virtual item during any routine, it will be activated and deserialized into memory. After the operation is completed and the item is no longer required, the item is serialized back to the file system and memory is freed by the JVM’s garbage collector. The application may be set to the virtual mode for the entire session, in between operator executions or just for a single operation. This procedure can be performed on-line and does not require a restart.

All functions of this tier are extensively used by the next higher business logic tier, but are not supposed to be used by the presentation tier directly.

**Business Logic Tier**. The business logic tier implements the use cases of a software application and ties together data and presentation tier. It controls the behaviour of the application at runtime, e.g. performs consistency checks of data items passed to operators and hosts the processing algorithms. Image and signal processing algorithms (operators) are the heart of the IQM operator framework. A workflow is a sequence of one or more operator executions and controlling them is essential to the stability of the application.

The abstract factory design pattern [37] is widely used in IQM, e.g. for the creation of processing tasks and operators. It facilitates the creation of objects without concrete application context and enables a more modular application architecture. Based on specific declarations or signatures, i.e. how many input parameters an algorithm takes or whether it is an image or signal processing algorithm, factories instantiate objects of the corresponding class and bind them to the application context at runtime. This enables loose coupling of client code and library, or framework, respectively. Alongside various registries and generic interfaces, this concept is of utmost importance for the operator plugin framework and hence for the extensibility of the software. The concept of operator plugins and how it is realized in IQM will be described in detail in section “Extensibility”.

**Presentation Tier**. A GUI is usually more attractive than a simple command-line interface and tends to lead to higher user acceptance [38]. Visual information representation is crucial to digital image processing, especially when it comes to the evaluation of different algorithms in a comparative manner. However, a convenient and user-friendly GUI is accompanied by significant effort in software engineering. Bad usability is one of the top reasons why the best applications are intended to fail acceptance in practice. Especially in (bio)medical imaging one is expecting good usability due to the fact that visual data can get extremely complex [39].

Nowadays, researchers use several kinds of operating systems in parallel and often require their tools being portable from one platform to the other. Users also expect software to integrate fully with the familiar appearance of their operating system. The Java Swing API provides a comprehensive toolkit for the construction of flexible GUIs and is available without additional libraries in the standard Java Runtime Environment (JRE). IQM’s GUI is based on Swing and is thus portable across any operating system capable of running the Java Virtual Machine (JVM). Custom components and widgets based on Swing can be developed on demand, preserving the mobility of the entire application package.

The presentation tier handles all user interaction on the GUI, e.g. drawing annotations on the image canvas. Furthermore, it triggers business logic processes and visualizes results after operator executions in the corresponding UI elements.

### Functional Architecture

Functional architecture can be viewed as the sum of components implementing functionality required for specific use cases. The functional components are not exclusively bound to a specific tier, but may span vertically over more tiers, see Fig. 3.

Over time, most software applications require new functionality to be added constantly. Modular software architectures enable a controllable development and administration of new functionality following state-of-the-art design patterns. The functional architecture of IQM comprises five modules:

- API
- APP (bootstrapper)
- CORE
- PLOT-OP-BUNDLE
- IMG-OP-BUNDLE

Deployment of the application as modules facilitates the exchange of single components, e.g. for updates.

**IQM API.** The application programming interface (API) of IQM contains a collection of all necessary interfaces, abstract implementations as well as events, emitter and listener interfaces, which are required for developing new functionality and plugins. This module contains the data model and interfaces to the main workflow control elements Tank and Manager. Furthermore, factory interfaces for the extensibility are specified in the API module. This property makes this module to the most important one in the entire IQM framework. Extensions to the API are occasionally made for new features, while downward compatibility is maintained.

Since version 3.1, IQM also provides an interface to the dynamical object-oriented scripting language Groovy [40]. With this new feature on board, users are able to develop re-usable processing routines within the IQM framework. The API is responsible for injecting required variables and dependencies into Groovy bindings, which are used to pass the application context to the script files.

**IQM APP.** This module is mainly a bootstrapper for the core module and responsible for initializing system and application properties. At startup, this module creates the application context in terms of building the GUI, searching for operator plugins and binding them to the core module.

**IQM CORE.** The core module is a concrete implementation of the interfaces and abstract methods provided by the IQM API. It hosts major parts of the GUI and processing framework. Long-running procedures are executed in the background and are hidden from the high-level user interface. The most important parts in this module are the registries and factories for operator plugins, which are used by the bootstrap module at startup in order to register operators included in the standard bundles and plugins.

**The Standard Operator Bundles.** Image and signal operators (algorithms) that are part of the standard application are bundled and deployed in two packages. The image operator bundle contains all image processing algorithms as well as their GUIs in order to adjust the operator’s parameters. Each operator owns a validation scheme, where the sources passed to the operator are checked prior to the execution. IQM’s image processing functionality is build mainly around the Java Advanced Imaging (JAI, available from https://java.net/projects/jai) library. This comprehensive image processing library provides functionality used in most of the image processing operators. It is also possible to integrate ImageJ plugins into the IQM framework, which complements the operators perfectly. On the other hand, the signal operator bundle contains algorithms which operate on (multiple) 1D time-dependent signals. Also, GUI and validation schemes are included in this bundle. Currently, 50 image and 16 signal operators are part of the standard bundles.

The idea behind creating two more comprehensive libraries instead of developing every operator in a single plugin was that we intend to deploy standard algorithms along the application. Highly customized operators may, however, be developed as plugins.

## Proof-of-Principle Analysis

In this section, we demonstrate the versatility of IQM’s image and signal processing capabilities using a real-world example. A standard use case consists of three steps: (i) loading or generating items such as images or signals, (ii) processing them using various algorithms, and (iii) saving the processed results or taking them as input for subsequent processing steps.

In this proof-of-principle analysis, a time-lapse video showing the movement and proliferation of cells was chosen for showcasing analysis with IQM. Since our focus is on the application of IQM, only basic information regarding the cells, their treatment and the recording of the video is given. The investigated cells originated from the human epidermoid carcinoma cell line A431, which is commercially available. The cells were defrosted and incubated using standard procedures. For microscopic observation, they were placed in a micro incubator, which was mounted on the microscope stage. During the recording, the cells were perfused with a nutrition solution providing physiological conditions. An optical transmission microscope was used to obtain a series of phase-contrast images through the viewing glasses of the micro incubator. Starting from *t* = 0 min, every five minutes an image was taken with a CCD camera and saved in JPEG format with a resolution of 1300 × 1030 pixels. In total 999 images were recorded, covering a total time of over 83 hours. Subsequently, all images were resized and merged to one single video file (AVI, no compression) with a resolution of 650 × 515 pixels and a frame rate of 10 fps.

**Figure 4:**
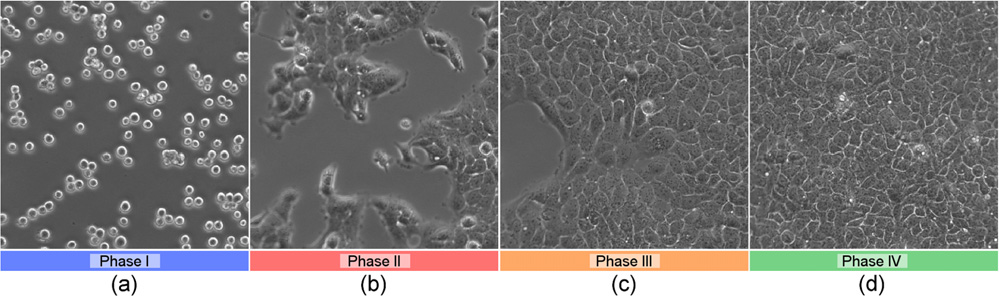
Frames of the Proof-of-Principle Analysis. Selected frames of the video in the proof-of-principle analysis. In the first part of the video ((a), phase I), the recorded cells from the epidermoid carcinoma cell line A431 move freely in the nutrition solution until they adhere to the viewing glass. In the second phase, more and more of the visible region is occupied due to cell proliferation ((b), phase II). One part of the recorded area stays blank for a longer time ((c), phase III), before this part is occupied as well and the whole recorded area is occupied by a complete monolayer of the cells ((d), phase IV).

In the therewith obtained time-lapse video, different phases of the cells under investigation are distinguishable, which are depicted by selected frames in Fig. 4:

- Phase I—Fig. 4(a)
  The first couple of frames show the solitary, unattached cells moving around rather quickly, i.e. they move freely in the nutrition solution, mainly because of the flow of the solution itself. In the end of this phase, the cells start to adhere to the viewing glass.
- Phase II—Fig. 4(b)
  After the cells have adhered to the viewing glass, proliferation starts and more and more of the visible region is occupied by cells and conglomerates of cells.
- Phase III—Fig. 4(c)
  The growth of the occupied area nearly stops. A part of the recorded area stays free of cells for some time. In the end of this phase, also this area is occupied.
- Phase IV—Fig. 4(d)
  In the last part of the video, the whole visible region stays occupied by a monolayer of the cells, still showing proliferation processes.

The aim of this proof-of-principle analysis was to answer the following questions:

- How long does it take until the cells adhere to the viewing glass (*t*_1_)?
- Starting from *t*_1_, how long does it take until a complete monolayer of cells is formed (*t*_2+3_)?

by applying IQM’s built-in methods without necessity of any programming, but only interacting with the GUI. To ensure reproducibility, the link to the video file and detailed steps of this example are given in IQM’s user guide, which can be downloaded from the project website.

In the following, the procedure used for analysis is summarized. Although the source video includes a total of 999 frames, the last cell phase IV already starts at about frame #600. Hence, we can omit the last 200 and load only *N* = 800 frames. Some of the video frames suffer from artifacts due to technical problems during recording. In these images, two distinct regions are distinguishable, which exhibit different intensities separated by a horizontal border. In a first step of the analysis, IQM was used to identify these frames by stack-processed evaluation of the mean grey values. Histogram normalization was applied to all images *I_i_*_=1…*N*_ of the video in order to eliminate differences in illumination. Then, absolute intensity difference images *J_j_*_=1…*N*-1_ = |*I_j_*_+1_ — *I_j_*| were computed.

**Figure 5:**
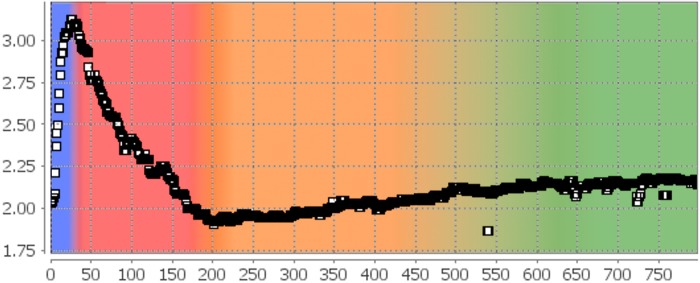
Skewness of the Difference Images in the Proof-of-Principle Analysis. From the histograms of the absolute image differences, statistical parameters like energy, entropy, skewness, and kurtosis were determined. The figure shows the plot of the skewness taken from the Viewport of IQM. Phases I (blue) and II (red) are clearly distinguishable: During phase I the skewness rises steeply reaching a maximum at the transition to phase II at about frame #20. During phase II, the skewness is decreasing and reaches a minimum at the transition to phase III (orange) at about frame #200. The transition from phase III to phase IV (green) could not be detected with this approach.

From the histograms of the resulting images *J*, the robust statistical parameters energy, entropy, skewness and kurtosis were calculated and plotted versus the image number *j*. All of them show distinct differences in their characteristics for phases I, II, and the combined phases III and IV. As an example, the obtained skewness is depicted in Fig. 5, the different phases are indicated with colors (blue: phase I, red: phase II, orange: phase III, green: phase IV). During phase I, the skewness is rising, reaching a maximum value just before the start of phase II at about frame #20, during which it is decreasing until phase III starts at about frame #200. No significant change of the curve’s characteristic is observable at the transition from phase III to phase IV. Energy and kurtosis show similar, entropy shows inverted behavior. Median filtering was applied to the four signals to eliminate outliers, then positions of local maxima and minima were identified. The mean values and the corresponding standard errors were calculated to obtain the length of phase I

$$t_{1}=(105\pm 25)\text{min}.$$

After this time the cells start to adhere to the viewing glasses. With the same method the length of phase II was found to be *t*_2_ = (920 ± 35) min.

**Figure 6:**
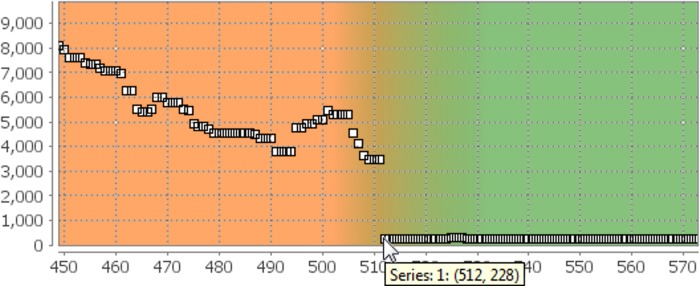
Transition from Phase III to Phase IV in the Proof-of-Principle Analysis. Region growing was used to determine the size of the area which was not occupied by the cells. The figure gives this size in pixels as a function of the frame number as it was displayed in the Viewport of IQM. The transition from phase III (orange) to phase IV (green) is identifiable as the frame at which the size of the spot reaches and stays at its minimum (frame #512).

However, the combined time of phases II and III, i.e. the time needed to create a full monolayer of cells in the investigated region, is more interesting. Hence, the time was determined when the transition from phase III to phase IV occured. Region growing was used to measure the size of the unoccupied spot in the original images *I* as a function of the frame number *i*. After applying a median filter to the obtained signal, the frame was identified, at which the whole observed region was occupied by a monolayer of the cells. The filtered signal is shown in Fig. 6. Thus, the time period was determined, after which the investigated area was fully occupied by a monolayer of the cells. With the found value of *t*_1+2+3_ = (2560 ± 25) min, the time period from the adhesion of the cells to a full monolayer was found to be

$$t_{2+3}=(2455\pm 50)\text{min}\approx (41\pm 1)\text{h}.$$

## Extensibility

Since a complete re-deployment of an entire application is rather impracticable for adding new functionality, modern sustainable software architectures support extensibility via modular components. Plugins are the most common way to extend applications without re-compiling.

IQM provides a plugin framework responsible for locating, loading and integrating the functionality of image and signal processing operators at runtime.

### Operator Plugin Framework

This section covers the following aspects: (i) the structure of the IQM operator plugin framework, (ii) the common structure of image and signal operators and (iii) the encapsulation of operators in a plugin. We illustrate the structure of an operator using an example implementation of a plugin, since plugins are designed to encapsulate operators, but first we introduce the framework, which will host the plugins at runtime.

**Figure 7:**
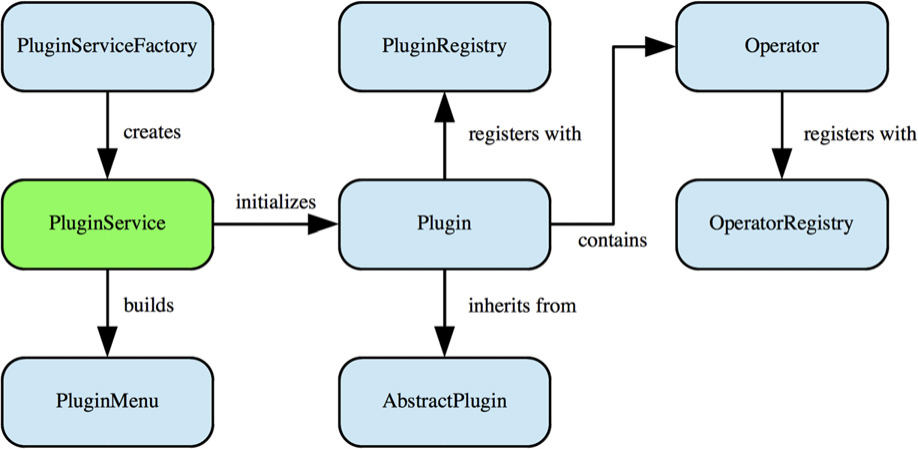
IQM Operator Plugin Framework. The operator plugin framework enables adding custom signal and image processing operators as plugins. The PluginService component manages the integration of the plugin into business logic and presentation layer. A plugin is registered with the PluginRegistry, the containing operator is registered with the OperatorRegistry. Thus, the operator factory is able to create instances of operators by querying the registry.

IQM’s operator plugin framework consists of four major parts: PluginService, PluginRegistry, OperatorRegistry, and the Plugin itself (see Fig. 7). The PluginService can be viewed as central hub in the plugin framework, which is responsible for recursively locating and loading plugins from the plugin directory to the classpath of the JVM. Furthermore, it calls the initialization method of the plugin interface which in turn performs a self-registration of the plugin in the PluginRegistry.

Each plugin inherits a property manager from the abstract implementation, which is responsible for reading plugin meta-data from an XML property file and locating resources such as menu icons in designated locations. The operator itself is registered with the OperatorRegistry separately.

**Figure 8:**
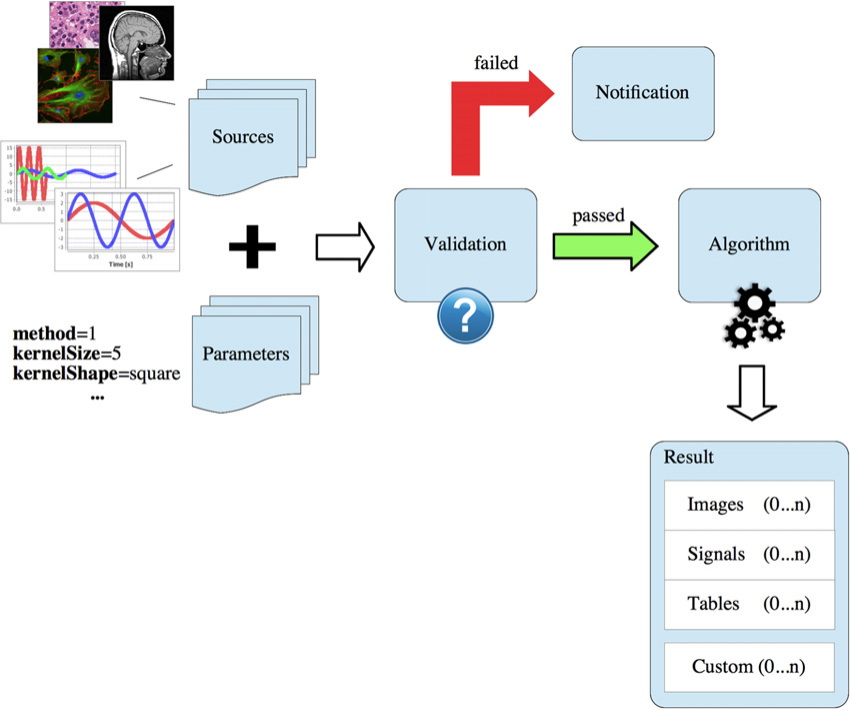
General Operator Execution Flow. Each operator (algorithm) may take heterogeneous sources and parameters as input and processes them to a multi-dimensional result. The result may contain multiple images, signals, or tables. Furthermore, custom output objects are also possible to be returned by an operator. The cardinality (0… *n*) denotes that the result may yield zero or more elements of each kind.

**Operator Structure**. Each operator consists of (i) a descriptor, (ii) a validator, (iii) the actual algorithm, and (iv) a GUI. An operator processes sources (images, signals, …) along a set of parameters to a result, see Fig. 8. A concrete definition of the operator in terms of unique name (for the registry), total number and types of sources (image, or signal), and valid ranges of parameter values is declared in the descriptor class. In the general setting of using operators via the GUI, sources and parameters are validated implicitly in the workflow. The operator validator contains custom rules for sources and parameters, e.g. checking a source image for a specific color or sample model, or checking the length of signals. Validation may be bypassed in some situations, where a processing chain clearly defines the inputs to the operators (e.g. in scripts or when cross-calling other operators). If the validation fails, detailed error messages are displayed, providing evidence of the underlying error. Otherwise, the algorithm is run with the specified parameters and sources. Sources are usually selected in the Manager, and parameters are conveniently set using the operator GUI. Once suitable parameters have been determined, they may be stored in a persistent template for subsequent processing.

Per definition, each operator is able to produce heterogeneous, multi-dimensional result objects: multiple images, signals or tables, see Fig. 8. For example, an image operator may deliver multiple sub-images (patches), accompanied by a set of statistical texture parameters for each item.

**Operator Plugin Example**. Standard operators are bundled and deployed with the application framework. Operator plugins are not designed to override, but to extend the existing functionality. The API module clearly defines interfaces that have to be implemented by classes in order to be used as plugins. Operators may be developed as single plugins and in this section we take a brief look under the hood of an operator plugin for a statistical texture descriptor. Detailed information on developing operator plugins can be found on the Wiki pages at the project’s website at https://sf.net/p/iqm/wiki. In this example, we implemented Local Binary Patterns (LBP [41]), a powerful statistical texture descriptor. The complete implementation of this plugin is available from the public source code repository at https://sf.net/p/iqm/code-0 as well as in S1 File. We will briefly introduce how the algorithm works and discuss the results it will produce. Since in (bio)medical imaging tissue samples from biopsies may also be considered as highly textured regions, we can describe the content by LBP pixel-wise (dense grid sampling).

**Operator Descriptor.** The operator descriptor defines an algorithm’s behaviour in the IQM framework. From a set of predefined values the operator type is assigned to the algorithm, which determines the context-aware activation and deactivation of the associated entry in the plugin menu. In this example, we define our algorithm to be an image operator, since the menu should be active exclusively if an image is loaded. The list of output types determines the multi-dimensional nature of the processing result, i.e. which data types the operator is able to produce.

Each operator is identified by a unique name (protocol-like string) which is declared as property in the descriptor. The descriptor defines the number of sources as well as the name, type, valid value ranges and default values of parameters, see Table 3 for the LBP operator. Parameters are usually defined using primitive data types such as INT, FLOAT, etc and are encapsulated in a parameter block object.

**Table 3:** Default Parameters and Valid Ranges for the LBP Operator.

**Parameter Name**	**Type**	**Default Value**	**Valid Range**
Neighbours *P*	INT	8	${\text{Valid}}_{P}=\left\{P\in {\mathbb{Z}}^{+}|1\leq P\leq 32\right\}$
Radius *R*	FLOAT	1.0	${\text{Valid}}_{R}=\left\{R\in {\mathbb{R}}^{+}|R\geq 1.0\right\}$
Smooth (Gaussian)	BOOLEAN	*false*	{*true* | *false*}
Kernel Size *k*	INT	3	${\text{Valid}}_{k}=\left\{2i+1\in {\mathbb{Z}}^{+}|i\in {\mathbb{N}}^{+},i\leq 50\right\}$
Cells *C*	INT	1	${\text{Valid}}_{C}=\left\{C\in {\mathbb{N}}^{+}|1\leq C\leq \max\left\{M,N\right\}\right\}$, where *M*, *N* are height and width of the image

Default parameters are set when the operator descriptor is created. The descriptor also checks for valid ranges on instantiation in defined sets, see last column.

The descriptor also registers the operator with the OperatorRegistry when IQM is starting up. Options passed to the registry comprise—among others—the class names of descriptor, validator, algorithm, GUI for the factories to instantiate objects of the corresponding classes at runtime.

**Validator.** The operator validator is responsible for checking integrity of sources passed to the operator. The LBP operator requires a single-band grey value image at position 0 of the source vector. First, the default validator checks whether there is an image available. The custom validator subsequently checks for the required color model.

**Algorithm.** The standard LBP algorithm proposed in [41] first thresholds a center pixel’s intensity *I_c_* with the intensities *I_p_* of the eight adjacent pixels and radius *R* = 1 in a 3-by-3 regular grid neighbourhood (*P* = 8). The center pixel is assigned a binary code, depending on the sampling pattern defined by *P* and *R*

$$LBP_{P,R}=\sum_{p=0}^{P-1}sign(I_{p}-I_{c}{)2}^{p}$$ (1)

where *sign*(·) designates the sign function

$$sign(I_{p},I_{c})=\{\begin{array}{ll} 1 & \text{if}I_{\text{c}}\geq I_{\text{p}}\\ 0 & \text{otherwise} \end{array}$$

The code in an 8-neighbourhood can take values from 0 to 255 (8bit) and may conveniently be treated as decimal number and visualized as discrete grey value in an LBP image. The LBP algorithm has been extended to use arbitrary neighbourhoods and radii [42], thus generalizing to 2^*P*^ possible binary codes. In order to display the codes > 2^8^, we scale the intensities between 0 and 255. A set of *P* “adjacent” pixels is sampled from a circle around a center pixel defined by radius *R* in regular angles. Bilinear interpolation is used for calculating the intensities of in-between pixel locations.

Rotation invariance [42] was added to the descriptor

$$LBP_{P,R}^{ri}=\min\{rot(LBP_{P,R},i)\;|\;i=0,\ldots ,P-1\}$$ (2)

where *rot*(·) is the rotation function used to minimize the binary code by a circular bit-wise right shift so that the most significant bit is 0.

Our implementation offers optional smoothing the image by convolution with a Gaussian prior to computing the descriptor in order to filter high frequencies, which most probably account for noise. Here we can also demonstrate, how integration of other operators is achieved: By calling *process()* on the work package instance, the task factory creates and executes a worker thread in the background, which runs the operator and produces a result.

An *M* × *N* image may also be partitioned in *C* × *C* cells, where for each cell the descriptor histogram is calculated and concatenated to form the final feature vector [43]. If *C* = 1, which is default, the descriptor is computed for the entire image at once. Additionally, we are interested in the raw binary codes for each pixel in a cell, so we put them in a table in order to store them later in a file.

LBP can for example be applied to describe the content of histopathological image data, where a distinction of malign and benign tissue regions is required. Supervised machine learning can be employed to learn the description of these regions in order to discriminate the tissue samples and highlight regions in a sample where probably malign tissue is present [44].

The rotation invariant texture measure is calculated for each pixel in a region. At the image borders, the $LBP_{P,R}^{ri}$codes were computed after extending the image using reflecting borders for ⌈*R*⌉ pixels. A histogram of the LBP codes forms the final descriptor of the region and the full image is described by concatenating the histograms. The descriptor can now be used for tissue or object classification.

**Figure 9:**
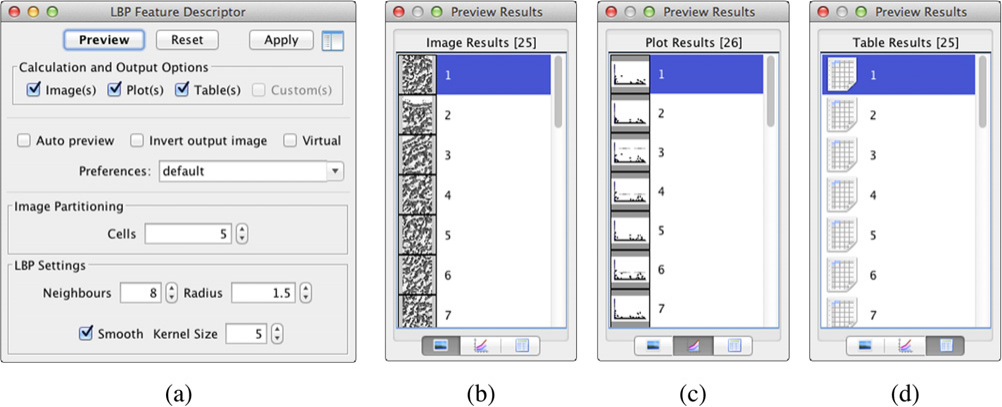
GUI and Multi-Result Dialog of the Local Binary Pattern Operator. The GUI of the LBP operator illustrated in (a) is used to set the parameters while performing integrity checks on the valid ranges defined in the descriptor. Calculation options indicate flags for the desired dimensions of the result object. “Auto preview” enables a quick examination while changing parameters. The output image may be inverted for better contrast and the operator may explicitly be set to the “virtual” mode prior to the execution. The multi-result dialog (b-d) hosts the preview results from an operator and lets the user choose whether all or just selected results should be kept for further processing.

**Operator and Result GUI.** The GUI is used for exploring the parameter space of an algorithm and to compute the responses in an interactive manner. Widgets like spinners or check boxes are used to set the parameters, see Fig. 9(a). A preview of the operator can be executed and shows the results. In this example, we let the algorithm compute a set of images, histograms and tables according to the number of cells. These results are displayed in the multi-result dialog after processing has finished, where each tab contains a list of the corresponding data type, see Fig. 9(b–d).

### Automatization via Scripting

**Figure 10:**
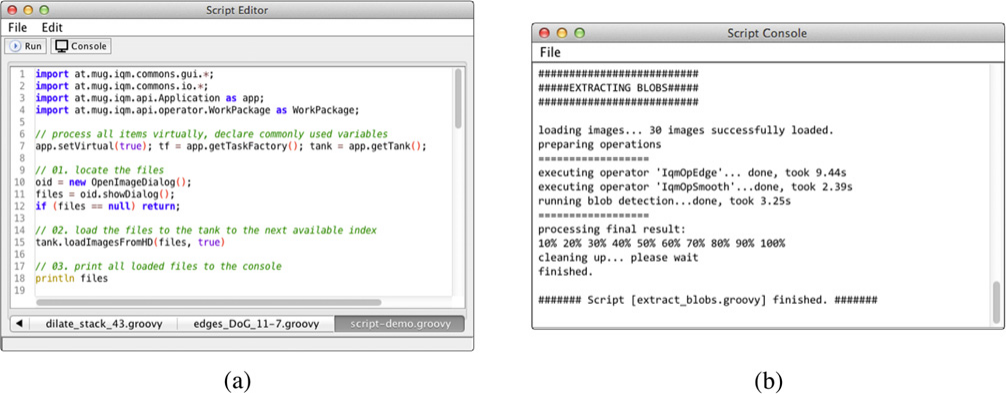
Groovy Script Editor and Console GUI. IQM provides a multi-tabbed, syntax-highlighting editor (a) for creating, editing and running Groovy scripts within the IQM framework. A console window (b) displays all output while the script runs and provides methods for saving the console output to a plain text file.

In daily routine some processing tasks often have to be executed frequently. Since setting parameters manually is tedious, scripts facilitate chaining of frequently used operations. Scripts are designed to ease the development of more complex workflows, i.e. execution sequences, using a dynamical high-level language. The development of custom scripts using Groovy language is available in IQM since version 3.1. Fig. 10(a) depicts the GUI of the script editor, a customized multi-tabbed text editor, which supports syntax highlighting. When running a script, the output is redirected to a custom console (Fig. 10(b)), where it may eventually be saved to a custom text file. Scripting in IQM ties together available methods for image and signal processing as well as machine learning and enables the development of advanced processing chains.

**Script Example.** In this section we want to demonstrate the scripting capability using a short example. We locate and load some images, perform two subsequent image operations and save the results as image sequence (running file index number) to a directory, see S1 Listing and S2 File of this article.

To start with, the application is set to virtual mode where each image is just loaded to memory for processing and resides on disk otherwise. A custom file dialog for opening supported image file types lets the user locate the files, which are about to be loaded to the Tank.

The first operator performs image denoising by convolving the image with an isotropic 2D Gaussian kernel. Therefore, the source images are taken from the current tank position and a default parameter set is created for the operator’s unique name, which is *IqmOpSmooth* in this case. Default parameters may be customized so that the work package contains all sources and parameters a processing task requires for execution. Resolving of “virtual” data items is performed implicitly by the IQM processing framework. The task factory creates a new task for serial processing, i.e. the images are processed with the same parameters in sequential order. The *execute()* command starts the processing and *get()* retrieves the multi-dimensional result. In this example we are just interested in the images, so we just take the first index (0) of the result and add them as interim result to the Tank.

The second operation takes the smoothed output images and extracts edges using the Difference of Gaussian (DoG) algorithm. Again, sources are added to the work package and default parameters are overridden. The second operation is executed in parallel using four threads, and the second interim result is added to the Tank. All processing steps of the script are subsequently available for closer examination in the Tank. The last part of the script saves the images as a sequence of PNG-encoded files to a directory, which the user selects via a file dialog.

This short example serves as illustration of the capabilities for automatization via scripts in IQM. Scripts are not limited to a pure orchestration of IQM standard operators, but rather let the user incorporate other libraries and operators from plugins as well.

### Compatibility and Interoperability

ImageJ, OpenCV [6] and MATLAB (The MathWorks, Inc., Massachussets, USA) are among others the most prominent software applications for image processing, and signal processing, respectively. Compatibility with established software packages is an important requirement and hence IQM makes use of existing interfaces for basic interoperability with these programs.

IQM packs an instance of ImageJ and they share a common classpath at runtime. Functions implemented as ImageJ plugins are therefore also available in the IQM framework. Moreover, a dedicated ImageJ instance can be launched, where image processing functions may be applied in the familiar ImageJ environment and the resulting image is then ported back to IQM for further processing. For example, a visualization of image stacks as 3D volumes is not part of IQM, but can be achieved with proper ImageJ plugins. Thus, IQM and ImageJ should not seen to be competing, but complementing each other.

The integration of powerful computer vision libraries like OpenCV in plain Java routines is possible since OpenCV version 2.4.4. But, in order to use their algorithms, OpenCV must be installed natively on the system and the Java bindings must be available on the IQM classpath. Though, a certain compromise between performance and portability of IQM must be considered: OpenCV implementations are usually in C/C++ and therefore faster than in plain Java, but the speed-up prohibits a simple transfer of the IQM package from one machine to another.

In addition to these two open source applications, IQM provides the ability to control a MATLAB instance via the open source library *matlabcontrol*, available from http://matlabcontrol.googlecode.com/. Besides reading proprietary *.mat files, MATLAB can be incorporated in processing routines in operator plugins and scripts.

State-of-the-art information technology enables the generation of a vast amount of data in almost all research areas. In order to cope with multi-dimensional data from different sources, machine learning (ML) has emerged as a common method in order to analyze complex patterns in digital imaging and signal processing. The advantage of ML over conventional pattern analysis is that ML systems are designed to increase performance with increasing experience, i.e. they learn from examples [45]. The popular Java open source machine learning suite WEKA [46] facilitates building complex algorithms including ML methods in IQM. Algorithms implemented in the WEKA library are accessible from IQM plugins and scripts.

## Discussion and Outlook

In this paper, we have presented IQM, a versatile and extensible open source software application written in Java. We provided insight into the functionality for image and signal analysis and gave an overview of the most important user interface components. IQM can be used as a single tool for sophisticated workflows involving both image and signal analysis without explicit programming skills, which was demonstrated in a proof-of-principle analysis. A comprehensive set of implementations among the available methods is dedicated to fractal image and signal analysis, which cannot be found in other free software packages. Another unique characteristic of IQM is the capability of on-demand virtual processing, where memory can be saved for actual processing tasks. Images can be annotated on multiple independent annotation layers using various ROI shapes in the image canvas.

The functional components (modules) based on the three-tier software architecture enable an arbitrary extension of IQM. Commonly and frequently used components may be added to the API module and are thus available to all future developments.

In section “Extensibility” two important concepts for adding functionality and automate workflows in IQM have been illustrated using example implementations: (i) the operator plugin framework and (ii) the Groovy scripting interface. The components of the Local Binary Pattern [41–43] texture descriptor plugin have been described in detail. The example script showed the convenient Groovy interface to IQM, where several operators are chained in a stack processing use case. Furthermore, the recent trend of using machine learning in digital image and signal analysis is supported via the integration of WEKA.

One of the main advantages of IQM lies in its portability across several operating systems. The binary application package can be run on the JVM and does not depend on pre-installed libraries. However, while better performance can be expected from applications compiled for specific platforms, they need to be installed and may depend on several third-party libraries. A minor drawback of the core image processing algorithms is the usage of JAI, since this library ran out of support in 2006. Nevertheless, there exists support for accelerated JAI algorithms on Solaris, Linux and Windows.

IQM is under active development and has been used in several research projects, e.g. in [3, 47–52]. Since IQM is released under the permissive GNU GPLv3 license, the source code and executables are available from the hosting platform sourceforge.net. Open source software offers great potential to reproducible research, since existing algorithms can be examined at source code level and new algorithms can be implemented in existing frameworks without the tedious development of frequently used functions such as locating, reading and writing supported file formats. Furthermore, the transfer to other programming languages can be achieved more easily. Open source software is more frequently used in medical science and bio-informatics [53], but may also co-exist with commercial software packages [54] in order to maximize productivity.

Future developments in IQM will focus on further interoperability with existing software, the implementation of new algorithms and the speedup of existing algorithms.

## Supporting Information

S1 File Implementation of the Local Binary Pattern texture descriptor operator within the IQM framework. This file contains the complete Java source code of the plugin described in section “Extensibility”.
(ZIP)

S2 File Implementation of the stack processing script. This file contains the source code for the example script for image stack processing discussed in section “Automatization via Scripting”.
(ZIP)

S1 Listing Groovy script example for image stack processing in IQM. This file contains the listing for the example script for image stack processing discussed in section “Automatization via Scripting”.
(PDF)
