# Supplementary figures and images for: IQM: An Extensible and Portable Open Source Application for Image and Signal Analysis in Java
Source: PLoS One. 2015 Jan 22;10(1):e0116329. doi: 10.1371/journal.pone.0116329 (PMC4303421; doi:10.1371/journal.pone.0116329)

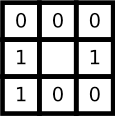

Supplement: S1 File — This file contains the complete Java source code of the plugin described in section “Extensibility”. (ZIP) [file pone.0116329.s001.zip › feat-lbp/src/main/resources/feat/lbp/icons/lbp-icon.png]

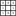

Supplement: S1 File — This file contains the complete Java source code of the plugin described in section “Extensibility”. (ZIP) [file pone.0116329.s001.zip › feat-lbp/src/main/resources/feat/lbp/icons/menu-item-disabled.png]
