# Supplementary material for: IQM: An Extensible and Portable Open Source Application for Image and Signal Analysis in Java
Source: PLoS One. 2015 Jan 22;10(1):e0116329. doi: 10.1371/journal.pone.0116329 (PMC4303421; doi:10.1371/journal.pone.0116329)
Supplement: S1 Listing — This file contains the listing for the example script for image stack processing discussed in section “Automatization via Scripting”. (PDF) [file pone.0116329.s003.pdf]

Listing S1. Groovy script example for image stack processing in IQM

```
import at.mug.iqm.commons.gui.*;
import at.mug.iqm.commons.io.*;
import at.mug.iqm.api.Application as app;
import at.mug.iqm.api.operator.WorkPackage as WorkPackage;

// process all items virtually, declare commonly used variables
app.setVirtual(true)
tf = app.getTaskFactory()
tank = app.getTank();

// 01. locate the files
oid = new OpenImageDialog();
files = oid.showDialog();
if (files == null) return;

// 02. load the files to the tank to the next available index
tank.loadImagesFromHD(files, true)

// 03. print all loaded files to the console
println files

// get the source images from the tank
srcImgs = tank.getTankDataAt(tank.getCurrIndex())

// 04. smooth all images using 2D Gaussian filter
wp = WorkPackage.create("IqmOpSmooth")
params = wp.getParameters()
wp.setSources(srcImgs) // get last loaded index
// override the default parameters
params.setParameter("Method", 1) // Gaussian
params.setParameter("KernelSize", 7) // 7x7 kernel
```

```
// process the stack in serial manner
task = tf.createSerialTask(wp, app.isVirtual())
task.execute()
// wait for the multi-dimensional result (List<List<IqmDataBox>>)
result = task.get();

// add images (index 0) to the tank
smoothImgs = result.get(0)
tank.addNewItems(smoothImgs)

// 05. detect edges using Difference of Gaussians
wp = WorkPackage.create("IqmOpEdge")
params = wp.getParameters()
wp.setSources(smoothImgs) // set the items to process
params.setParameter("Method", 7) // DoG option
params.setParameter("KernelSize1", 3) // 3x3 kernel
params.setParameter("KernelSize2", 5) // 5x5 kernel
params.setParameter("ResultOption", 1) // normalize

// process the stack in parallel using 4 threads
task = tf.createParallelTask(wp, app.isVirtual(), 4)
task.execute()
// wait for the multi-dimensional result (List<List<IqmDataBox>>)
result = task.get()

// add images (index 0) to the tank
edgeImgs = result.get(0)
tank.addNewItems(edgeImgs)

// 06. store images as sequence to custom location
sisd = new SaveImageSequenceDialog()
target = sisd.showDialog()
```

```
if (target == null) return;

// 07. write the image files
ifw = new ImageFileWriter(target, edgeImgs, "PNG", "png", ↘
    →ImageFileWriter.MODE_SEQUENCE)
ifw.writeSequence()
println "Image␣sequence␣saved␣to␣[" << target << "]."
return;
```
